# Patients With Type 2 Diabetes Are at Greater Risk of Developing New Hypertension and Chronic Kidney Disease Following COVID-19

**DOI:** 10.1155/jdr/8816198

**Published:** 2025-06-04

**Authors:** Justin Y. Lu, Shiv Mehrotra-Varma, Stephen H. Wang, Montek Singh Boparai, Sonya Henry, Jai Mehrotra-Varma, Tim Q. Duong

**Affiliations:** ^1^Department of Radiology, Albert Einstein College of Medicine and Montefiore Medical Center, New York, USA; ^2^Department of Surgery, Beth Israel Deaconess Medical Center and Harvard Medical School, Boston, Massachusetts, USA; ^3^Center for Health & Data Innovation, Albert Einstein College of Medicine and Montefiore Medical Center, New York, USA

**Keywords:** acute kidney injury, cytokine storm, postacute sequelae of SARS-CoV-2 infection (PASC), renal failure, Type 2 diabetes mellitus

## Abstract

**Background:** The purpose of this study was to test the hypothesis that COVID-19 status increases the incidence of new hypertension (HTN) and chronic kidney disease (CKD) in patients with Type 2 diabetes (T2D).

**Methods:** This retrospective study consisted of 46,448 patients with T2D from the Montefiore Health System in the Bronx (3/01/2020–7/01/2023), of which 13,801 had a positive COVID-19 test on record. Contemporary controls included those hospitalized for other lower respiratory tract infections (LRTIs) (*n* = 1638) and nonhospitalized patients without COVID-19 or LRTI (*n* = 31009). Outcomes were assessed at follow-up (2 months to 3 years) relative to baseline. Adjusted hazards ratios (aHRs) with 95% confidence interval (CI) were computed.

**Results:** The cumulative incidences of HTN (22.32% vs. 9.13%, *p* < 0.001) and CKD (6.20% vs. 2.03%, p <0.001) were significantly higher in nonhospitalized COVID-19 compared to non-COVID-19 patients, but not between patients hospitalized for COVID-19 and LRTI (*p* > 0.05). Nonhospitalized COVID-19 patients had higher risk of developing HTN compared to non-COVID patients during all follow-up (aHR 1.99, 95% CI [1.54, 2.57], *p* < 0.001), but hospitalized COVID-19 patients had similar risk of developing HTN relative to patients hospitalized for LRTI (aHR 1.26 [0.70, 2.27], *p* = 0.441). Nonhospitalized COVID-19 patients had higher risk of developing CKD compared to non-COVID patients during all follow-up (aHR 2.09 [1.69, 2.76], *p* < 0.001), but hospitalized COVID-19 patients had similar risk of developing CKD relative to patients hospitalized for LRTI (aHR 0.96 [0.79, 1.36], *p* = 0.131).

**Conclusions:** T2D patients with COVID-19 were at higher risk of developing new disorders compared with COVID-19-negative controls and were at similar risk compared with those hospitalized for other LRTIs.

## 1. Background

Patients with pre-existing Type 2 diabetes (T2D) who are infected by SARS-CoV-2 have been shown to experience more severe acute COVID-19 outcomes, including a heightened risk of critical illness and mortality, compared to individuals without pre-existing T2D [1–12]. Some COVID-19 patients with pre-existing T2D are also at higher risk of morbidities such as cardiovascular dysfunction [13, 14] and acute kidney injury [15–19] during acute COVID-19. Acute COVID-19 related systemic hypoxia, hyperinflammatory response, and medications used to treat acute COVID-19 could further contribute indirectly to blood pressure and renal dysfunction [20–23]. Together, these acute events may increase the downstream risk of developing new-onset hypertension (HTN) [14], chronic kidney disease (CKD) [24], and other complications long after patients recover from acute COVID-19 [25]. However, the lasting consequences for COVID-19 survivors with pre-existing T2D with respect to the risk of developing new-onset HTN and CKD are unknown. Given the large number of individuals with T2D affected by COVID-19, these sequelae could pose a significant public health burden. Thus, it is important to determine whether SARS-CoV-2 infection increases the risk of new incident HTN and CKD in individuals with pre-existing T2D and identify risk factors associated with the development of new HTN and CKD as doing so could provide valuable insights for physicians for long term monitoring and management of patients with T2D who had COVID-19.

The goal of this study was to examine the incidence of newly diagnosed HTN and CKD up to three-years following SARS-CoV-2 infection and to identify risk factors associated with the development of these disorders. Comparisons were made against patients with T2D without a history of COVID-19 and those who had been hospitalized for other lower respiratory tract infections (LRTIs) as controls.

## 2. Methods

### 2.1. Data Source

This retrospective study was approved by the Einstein–Montefiore Institutional Review Board (#2021-13658). All polymerase chain reaction (PCR) test-confirmed COVID-19 patients and non-COVID-19 patients within the Montefiore Health System (which consists of multiple hospitals located in the Bronx and the lower Hudson Valley and Westchester County) from March 1, 2020, to July 1, 2023, were included in this retrospective analysis. Data were extracted as described previously [7, 8]. The follow-up time was up to 3 years.

**Figure 1:**
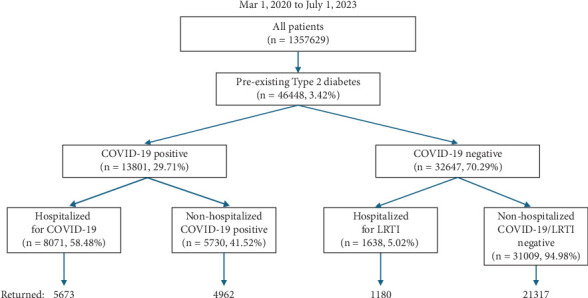
Flowchart illustrating patient selection.

The date of a patient's first positive COVID-19 or LRTI result or the first visit to the Montefiore Health System after March 1, 2020, was used as the index date for each cohort. There were 1,357,629 unique patients in the Montefiore Health System over the study period.  Figure 1 shows the patient selection flowchart. Only patients who had pre-existing T2D per ICD-10 code (E10.x, Table S1) were included. Patients were then stratified by COVID-19 status. For the COVID-19 cohort, patients were subdivided into nonhospitalized and hospitalized groups. For the non-COVID-19 cohort, patients were subdivided into a nonhospitalized group and a group hospitalized for other reasons, of which included LRTI. LRTI included influenza, bronchiolitis, pneumonia, and bronchitis (ICD-10 codes J09-22, Table S2). If a patient had any positive COVID-19 or LRTI test within 2 weeks of hospitalization, they were categorized in the hospitalized cohorts.

### 2.2. Patient Variables

Demographics, baseline comorbidities, and newly developed conditions at follow-up were collected. Demographic data included age, sex, race, and ethnicity. Race and ethnicity were based on patient self-identification and categorized as White, Black, Asian, or Others (comprising non-Hispanic patients indicating race as multiple selected, American Indian or Alaska Native, or some other race) and Hispanic or non-Hispanic. Comorbidities included HTN, CKD, cardiovascular disease, asthma, chronic obstructive pulmonary disease (COPD), and obesity. Newly developed HTN and CKD were tabulated. Laboratory data collected from hospitalized patients included HbA1c, averaged across 1-year before the index date (baseline). All patients analyzed had no missing data, with exception of HbA1c (*n* is listed in Table 1, and not analyzed as part of statistical modeling).

### 2.3. Outcomes

The primary outcome was incidence of newly developed diagnosis of HTN or CKD at all follow-up from at least 2 months post index date and up to July 1, 2023, using relevant ICD-9 and ICD-10 codes.

### 2.4. Statistical Analysis

All statistical analyses were performed on Python package Statsmodels. Group comparison for categorical variables was performed using the *χ*^2^ test, and the independent *t*-test for continuous variables. Incidence of HTN and CKD at follow-up were estimated using Kaplan–Meier curves and log-rank hazard ratios (HRs) with 95% confidence interval (CI). Cumulative incidence functions were calculated, and multivariable Cox proportional hazard model was used to estimate HR and 95% CI, adjusting for all demographics and comorbidities. Patients were censored at death or their last follow up within the health system. *p* values less than 0.05 were considered statistically significant. The proportional hazard assumption was checked using the Schoenfeld residuals, and it did not show any time varying effect, confirming the validity of using Cox regression model.

Given the large differences in patient characteristics between cohorts, we performed matching as a sensitivity analysis. We used 1:1 nearest neighbor propensity-score matching without replacement, with the propensity score estimated using logistic regression of virus type on age, sex, and ethnicity. After matching, 1180 hospitalized COVID-19 patients were successfully matched to 1180 hospitalized LRTI patients, and 4962 nonhospitalized COVID-19 patients were successfully matched to 4692 nonhospitalized non-COVID patients.

## 3. Results

There were 46,448 patients with pre-existing T2D in the Montefiore Health System, of which 13,801 (29.71%) tested positive for COVID-19 (Figure 1). Of the patients with COVID-19, 8071 patients were hospitalized, of which 5673 returned to our health system, and 5730 patients were not hospitalized, of which 4962 returned. In the non-COVID cohort, 1638 patients were hospitalized for LRTI of which 1180 returned, and 31,009 patients were not hospitalized and did not have LRTI of which 21,317 returned. Hospitalized COVID-19 patients had a follow up time of 419 ± 277 days (mean ± standard deviation), and hospitalized LRTI had a follow up time of 354 ± 244 days. Nonhospitalized COVID-19 patients had a follow up time of 357 ± 242 days, and nonhospitalized non-COVID patients had a follow up time of 492 ± 304 days.

Table 1 summarizes the characteristics of all patients with T2D with and without COVID-19, stratified by hospitalization status, who returned to our health system. Compared to the hospitalized LRTI cohort, patients hospitalized for COVID-19 were younger (64.08 ± 14.99 vs. 69.08 ± 13.81 years, *p* < 0.001), had lower prevalence of pre-existing cardiovascular disease (40.73% vs. 46.69%, *p* = 0.005), asthma (27.12% vs. 32.37%, *p* = 0.004), and COPD (20.67% vs. 32.29%, *p* < 0.001), and lower HbA1c levels within 1-year pre-index (7.75 ± 2.19 vs. 7.13 ± 1.81, *p* < 0.001).

Compared to the nonhospitalized non-COVID cohort, the nonhospitalized COVID-19 cohort was younger (60.03 ± 14.01 vs. 61.28 ± 16.96), more likely to be Hispanic (44.50% vs. 35.99%, *p* < 0.001), Black (34.70% vs. 31.49%, *p* < 0.001), and of race Other (43.67% vs. 38.19%, *p* < 0.001), less likely to be male (38.09% vs. 50.17%, *p* < 0.001) and White (10.18% vs. 12.28%, *p* = 0.003), and had higher prevalence of pre-existing HTN (81.66% vs. 68.16%, *p* < 0.001), CKD (28.68% vs. 19.89%, *p* < 0.001), cardiovascular disease (21.44% vs. 11.36%, p <0.001), asthma (29.16% vs. 13.04%, *p* < 0.001), COPD (11.89% vs. 6.09%, *p* < 0.001), obesity (46.09% vs. 27.91%, *p* < 0.001), and lower HbA1c levels within 1-year preindex (7.40 ± 1.81 vs. 7.98 ± 2.16, *p* < 0.001).

**Figure 2:**
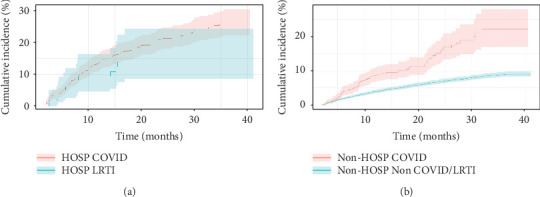
Cumulative incidence of new hypertension (HTN) for all patients with T2D who returned to our health system with (a) hospitalized COVID versus LRTI and (b) nonhospitalized COVID versus non-COVID/LRTI.

Figure 2 shows the cumulative incidence for HTN stratified by hospitalization status from 2 months to 3-years post-index date. The cumulative incidence of HTN among the hospitalized COVID-19 cohort was not significantly different from that of the hospitalized LRTI cohort (26.13% vs. 15.67%, *p* = 0.137). The cumulative incidence of HTN among the nonhospitalized COVID cohort was significantly higher than that of the non-COVID cohort (22.32% vs. 9.13%, *p* < 0.001).

Cox proportional hazard models were performed to examine the effect of COVID-19 relative to other potential risk factors for incident HTN using adjusted HRs for all patients without preexisting HTN stratified by hospitalization status (Table 2). Patients hospitalized for COVID-19 had similar risk of developing HTN during (aHR 1.26, 95% CI [0.70, 2.27], *p* = 0.441) relative to patients hospitalized for LRTI. By comparison, nonhospitalized patients with COVID-19 had greater risk of developing HTN (aHR 1.99, 95% CI [1.54, 2.57], *p* < 0.001) relative to non-COVID patients. Older (aHR 1.02, 95% CI [1.01, 1.02], *p* < 0.001) and male (aHR 1.39, 95% CI [1.19, 1.64], *p* < 0.001) patients had greater risk of developing HTN.

**Figure 3:**
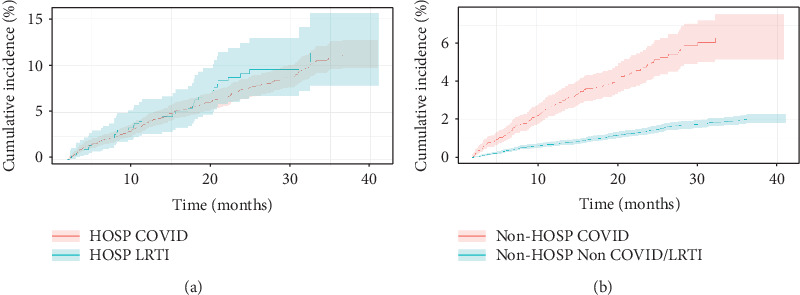
Cumulative incidence of new chronic kidney disease (CKD) for all patients with T2D who returned to our health system with (a) hospitalized COVID versus LRTI and (b) nonhospitalized COVID versus non-COVID/LRTI.

Figure 3 shows the cumulative incidence for CKD stratified by hospitalization status from 2 months to 3-years post-index date. The cumulative incidence of CKD among the hospitalized COVID-19 cohort was not significantly different from that of the hospitalized LRTI cohort (11.27% vs. 11.31%, *p* = 0.786). The cumulative incidence of CKD among the nonhospitalized COVID cohort was significantly higher than that of the non-COVID cohort (6.20% vs. 2.03%, *p* < 0.001).

Cox proportional hazard regressions were performed to examine the effect of COVID-19 relative to other potential risk factors for incident CKD using adjusted HRs for all patients without preexisting CKD stratified by hospitalization status (Table 3). Patients hospitalized for COVID-19 had similar risk of developing CKD at follow-up (aHR 0.96, 95% CI [0.79, 1.36], *p* = 0.131) relative to patients hospitalized for LRTI. By comparison, nonhospitalized patients with COVID-19 had greater risk of developing CKD at follow-up (aHR 2.09, 95% CI [1.69, 2.76], *p* < 0.001) relative to non-COVID patients. Older (aHR 1.04, 95% CI [1.03, 1.05], *p* < 0.001) and male (aHR 1.24, 95% CI [1.52, 2.23], *p* < 0.001) patients had greater risk of developing CKD, as well has patients with preexisting HTN (aHR 2.89, 95% CI [1.85, 6.01], *p* < 0.001) and cardiovascular disease (aHR 1.50, 95% CI [1.01, 2.22], *p* = 0.020).

As a sensitivity analysis, we also matched cohorts based on age, sex, and ethnicity and to examine the effect of COVID-19 relative to other potential risk factors for incident HTN and CKD for all patients without the respective preexisting condition stratified by hospitalization status (Table S3).  Table 4 shows that patients hospitalized for COVID-19 had similar risk of developing HTN at follow-up (aHR 1.37, 95% CI [0.76, 2.33], *p* = 0.398) relative to patients hospitalized for LRTI. By comparison, nonhospitalized patients with COVID-19 had greater risk of developing HTN at follow-up (aHR 1.74, 95% CI [1.42, 2.19], *p* < 0.001) compared to non-COVID patients. For CKD, patients hospitalized for COVID-19 had similar risk of developing CKD at follow-up (aHR 0.85, 95% CI [0.63, 1.15], *p* = 0.187) relative to patients hospitalized for LRTI, while nonhospitalized patients with COVID-19 had greater risk of developing CKD at follow-up (aHR 2.02, 95% CI [1.57, 2.60], *p* < 0.001) relative non-COVID patients.

Table S4 shows the new incidence of HTN and CKD and their HRs for developing new HTN and CKD due COVID-19 status were computed for three follow-up periods (2–6, 6–12, 12–36 months post index date). There were no significant differences across all time points for hospitalized cohort, but there were significant differences across all time points (except the 2–6 month follow-up for incident HTN and HR for new CKD) for nonhospitalized cohort. *p* values became smaller at the later time points.

The new incidence of HTN and CKD were also computed for three time periods (2020–2021, 2021–2022, and 2022–2023) (Table S5). There were no significant differences between patients hospitalized for COVID-19 and patients hospitalized for LRTI across all three time periods for both HTN and CKD, but there were significant differences between nonhospitalized patients with COVID-19 and patients not hospitalized for COVID or LRTI across all three time periods for both HTN and CKD.

## 4. Discussion

This study characterized individuals with pre-existing T2D infected by SARS-CoV-2 and identified risk factors associated with the development of new HTN and CKD up to 3 years postinfection in a diverse underserved inner-city population in the Bronx. The key findings are as follows: (i) The cumulative incidences of new HTN and CKD were significantly higher in COVID-19 patients compared to non-COVID-19 patients, with patients hospitalized for COVID-19 having higher cumulative incidences of new HTN and CKD; (ii) COVID-19 status was significantly associated with new incident HTN and CKD after adjusted for competing factors; (iii) however, when compared with patients hospitalized for LRTI, patients hospitalized for COVID-19 have similar adjusted risks for new incident HTN and CKD. Nonetheless, the sheer numbers of individuals hospitalized for COVID-19 are also likely of public health concern.

### 4.1. HTN

Although the association between T2D and new incident HTN is known [26], this is the first study to report the rates of newly developed HTN in individuals with T2D 3 years post SARS-CoV-2 infection. Nonhospitalized COVID-19 survivors with T2D were at higher risk of developing new-onset HTN 3 years postinfection compared to only T2D patients without COVID-19 and without LRTI. HTN is associated with COVID-19 disease severity (COVID-19 hospitalization as a surrogate), further supporting the notion that COVID-19 contributes to the pathogenesis of HTN. By comparison, a recent study found 20.6% of all (not just T2D) patients hospitalized with COVID-19 and 10.85% of all nonhospitalized patients with COVID-19 developed new HTN at 6 months postinfection [14]. Our new incident HTN were comparatively higher as expected due to T2D comorbidities, despite averaging over a longer pandemic period (which likely consisted of fewer severe COVID-19 cases on average), underscoring the greater new incident of HTN in T2D patient population.

In the nonhospitalized COVID-19 cohort, the difference in new incident HTN grew progressively larger with time, suggesting that a latent and long-lasting effects of COVID-19 toward worsening of BP, which may require longer follow-up monitoring. Although new incident HTN for hospitalized COVID-19 patients did not reach statistical significance compared to that of hospitalized LRTI patients, new incident HTN exhibited a higher incidence of new HTN at late follow-up, again suggesting the possibility of more latent effects from COVID-19 on the development of HTN compared to other pulmonary infections. Moreover, the sheer numbers of individuals hospitalized for COVID-19 could post a significant public health concern. Our findings contribute to the growing list of newly reported disorders associated with long-term COVID-19 effects as documented in the literature [8, 13–15, 18, 27].

Given that HTN is a major risk factor of worse acute COVID-19 outcomes [2, 28, 29], it is not surprising that some COVID-19 patients could develop new HTN post-infection as there could be a bidirectional interaction. The mechanisms underlying how SARS-CoV-2 triggers new-onset HTN are unknown. It is possible that SARS-CoV-2 causes cardiac and blood pressure dysregulation as there is evidence that SARS-CoV-2 directly infects cardiac cells via the ACE2 receptors [30]. Acute COVID-19 disease may also stimulate the renin–angiotensin–aldosterone system (RAAS), and persistent activation of RAAS and/or endothelial injury has been reported among patients with COVID-19; both are associated with blood pressure elevation [31, 32]. Furthermore, severe COVID-19 infection, with systemic hypoxia, acute respiratory distress, hypercoagulation, sepsis, inflammation, metabolic stress, and cytokine storms, collectively strains the cardiovascular system, potentially causing blood pressure dysregulation [2, 20, 21, 28, 29]. Other factors contributing to the development of HTN following COVID-19 may include the effects of isolation, psychosocial stress, reduced physical activity, unhealthy diet, weight gain, and transient difficulty in assessing care (including diabetic care) during the pandemic [22]. Patients with pre-existing T2D have an increased risk of developing cardiovascular and noncardiovascular hospitalizations compared to those without T2D [33]. In short, the interplays between SARS-CoV-2 infection and the emergence of HTN involves direct viral impacts on the cardiovascular system, exacerbation of existing conditions, and pandemic circumstances that disrupt healthy lifestyle and access to care.

### 4.2. CKD

Similarly, although the association between T2D and new incident CKD is well-documented [34, 35], this is the first study to report a three-year cumulative incidence of new CKD in patients with T2D who had contracted COVID-19. Patients with COVID-19 exhibited increased cumulative incidence and risk of new CKD, with the highest seen among hospitalized patients at 11.27%, indicating an association with COVID-19 disease severity. There are no similar studies available with which to compare. One report found that the prevalence of CKD in individuals with T2D was 27% [34]. While comparison needs to be made with caution due to differences in study duration, study design, and patient populations, our finding suggests unusually high new CKD incidence in T2D patients post COVID-19 infection.

The difference in new incident CKD also grew progressively larger with time, suggesting latent and long-lasting effects of COVID-19 toward worsening of BP, and that longer follow-up care might be needed. Again, the contribution of COVID-19 toward development of CKD remains likely as the overall incidence of CKD was significantly higher in the ambulatory COVID-19 cohort than the non-COVID-19 cohort. Among hospitalized patients, there were no significant differences in incidence of CKD.

Given that CKD is a major risk factor of worse acute COVID-19 outcomes [2, 28, 29], it is not surprising that some COVID-19 patients could develop new CKD post-infection as there could also be a bidirectional interaction. The mechanisms underlying how SARS-CoV-2 triggers new-onset CKD are unknown. It is possible that SARS-CoV-2 causes renal dysregulation as there is evidence that SARS-CoV-2 directly infects renal cells via the ACE2 receptors. AKI is frequently observed in acute COVID-19 [13, 17, 19, 36–42], and some patients who experience AKI during acute COVID-19 would go on to develop new CKD. Furthermore, severe COVID-19 infection, with systemic hypoxia, acute respiratory distress, hypercoagulation, sepsis, inflammation, metabolic stress, and cytokine storms, collectively strains the cardiovascular system, potentially causing persistent kidney dysfunction. Similarly, pandemic circumstances include the effects of psychosocial stress, and altered lifestyle and transient difficulty in assessing care, among others, could also contribute to higher incident CKD.

### 4.3. Risk Factors

Using statistical modeling, COVID-19 status was found to be a significant risk factor for developing new HTN and CKD in ambulatory patients with pre-existing T2D after adjusting for covariates. HRs were about 2 and were comparable to, or even higher than, HRs due to major comorbidities. The significant risk factors for both HTN and CKD were largely similar, and they included age, male sex, and several common comorbidities. These findings are not surprising given existing literature regarding risk factors for HTN and CKD [43–46]. Our modeling also showed that advanced age was an independent risk factor for the development of HTN or CKD as expected [47–49].

It is worth noting that there is a known link between HTN and CKD [50]. Indeed, we observed higher percentages of patients with both new HTN and CKD compared to perspective controls, suggesting that the interplays between SARS-CoV-2 infection and existing T2D metabolic disorder could put T2D patients with COVID at higher risks of developing both sequelae in general.

In contrast, severe (hospitalized) COVID-19 patients did not have an increased risk of new onset HTN or CKD when compared to patients hospitalized for LRTI, suggesting that the long-term systemic effects of severe COVID-19 may be comparable to that of severe influenza or bacterial pneumonias and/or that hospitalized patients had more consistent follow-up. Nonetheless, the sheer numbers of individuals hospitalized by COVID-19 are likely nonetheless of public health concern.

### 4.4. Limitations

There are several limitations to our study. First, this retrospective study came from a single health system with multihospitals in the Bronx. The patient population in the Bronx is diverse and has high proportions of minorities, which may not be generalizable to other less diverse population cohorts. Multicenter and prospective studies are needed to validate these findings and achieve broader generalization. We did not investigate the duration or severity of diabetes, medication-adherence, frequency of physician visits, or other healthcare behaviors which may have influenced outcomes. We did not study patients with pre-existing T1D as these have been reported previously [51]. It is possible some patients might have previously undiagnosed HTN and CKD prior to COVID-19, though we anticipate the rate of underdiagnosis to be small and similar between groups. As with any retrospective study, there could be unintended patient selection bias and confounds.

## 5. Conclusions

Patients with pre-existing T2D who survive COVID-19 infection have higher risk of developing new HTN and CKD up to 3 years postinfection. COVID-19 may have latent effects of developing new HTN and CKD compared to other LRTIs. Identification of risk factors associated with development of new-onset disorders could improve long term monitoring and management of patients after COVID-19 infection among patients with T2D.

## Figures and Tables

**Table 1:** Demographics and pre-existing comorbidities at index date for hospitalized COVID-19 versus hospitalized LRTI and nonhospitalized COVID-19 versus nonhospitalized LRTI. *p* value < 0.05 was considered significant between hospitalized COVID+ and LRTI patients and nonhospitalized COVID-19 patients and nonhospitalized COVID-19/LRTI negative patients. SD: standard deviation.

	**Hosp COVID-19** **N** = 5673	**Hosp LRTI** **N** = 1180	**p** ** value**	**Non-hosp COVID-19** **N** = 4962	**Non-Hosp COVID-19/LRTI negative** **N** = 21317	**p** ** value**
Demographics *N* (%)						
Age, mean (± SD)	64.08 (14.99)	69.08 (13.81)	< 0.001	60.03 (14.01)	61.28 (16.96)	0.001
Male	2675 (47.14%)	556 (47.12%)	0.991	1890 (38.09%)	10695 (50.17%)	< 0.001
Hispanic	2414 (42.54%)	554 (46.95%)	0.006	2208 (44.50%)	7671 (35.99%)	< 0.001
White	643 (11.33%)	123 (10.42%)	0.394	505 (10.18%)	2617 (12.28%)	< 0.001
Black	2124 (37.43%)	442 (37.46%)	0.982	1722 (34.70%)	6713 (31.49%)	< 0.001
Other	2480 (43.71%)	543 (46.02%)	0.157	2167 (43.67%)	8141 (38.19%)	< 0.001
Pre-existing comorbidities *N* (%)						
Hypertension	5009 (88.28%)	1080 (91.53%)	0.002	4052 (81.66%)	14530 (68.16%)	< 0.001
CKD	2869 (50.56%)	641 (54.32%)	0.021	1423 (28.68%)	4239 (19.89%)	< 0.001
Cardiovascular disease	2311 (40.73%)	551 (46.69%)	< 0.001	1064 (21.44%)	2421 (11.36%)	< 0.001
Asthma	1539 (27.12%)	382 (32.37%)	< 0.001	1447 (29.16%)	2779 (13.04%)	< 0.001
COPD	1173 (20.67%)	381 (32.29%)	< 0.001	590 (11.89%)	1299 (6.09%)	< 0.001
Obesity (or BMI > 30)	2575 (45.38%)	504 (42.71%)	0.099	2287 (46.09%)	5949 (27.91%)	< 0.001
1-year preindex HbA1c, mean (± SD)	7.75 (2.19) *n* = 3110	7.13 (1.81) *n* = 534	< 0.001	7.40 (1.81) *n* = 1004	7.98 (2.16) *n* = 3408	< 0.001

**Table 2:** Hazard ratios for newly diagnosed HTN among patients with T2D for (a) hospitalized COVID versus LRTI and (b) nonhospitalized COVID versus non-COVID/LRTI during 3-year follow-up.

	**(A) Hosp COVID-19 vs. Hosp LRTI**			**(B) Non-hosp COVID vs. non-COVID/LRTI**		
	**HR**	**95% CI**	**p** ** values**	**HR**	**95% CI**	**p** ** values**
COVID positive status	1.26	[0.70, 2.27]	0.441	1.99	[1.54, 2.57]	< 0.001
Age (years)	1.01	[0.99, 1.02]	0.097	1.02	[1.01, 1.02]	< 0.001
Male	1.30	[0.91, 1.85]	0.152	1.39	[1.19, 1.64]	< 0.001
Black vs. White	1.00	[0.58, 1.71]	0.995	1.10	[0.83, 1.43]	0.452
Other vs. White	0.97	[0.56, 1.68]	0.918	0.97	[0.75, 1.25]	0.804
Hispanic vs. non-Hispanic	0.83	[0.56, 1.22]	0.334	0.95	[0.84, 1.09]	0.473
CKD	1.21	[0.81, 1.80]	0.340	1.12	[0.60, 2.10]	0.719
Cardiovascular disease	1.27	[0.84, 1.93]	0.252	1.05	[0.50, 2.21]	0.902
Asthma	0.88	[0.54, 1.40]	0.586	1.07	[0.68, 1.67]	0.770
COPD	1.04	[0.62, 1.74]	0.876	0.87	[0.36, 2.10]	0.752
Obesity (or BMI > 30)	1.04	[0.72, 1.49]	0.849	0.58	[0.39, 0.88]	0.009

**Table 3:** Hazard ratios for newly diagnosed CKD among patients with T2D for (a) hospitalized COVID versus LRTI and (b) nonhospitalized COVID versus non-COVID/LRTI during 3-year follow-up.

	**(A) Hosp COVID-19 vs Hosp LRTI**			**(B) Non-hosp COVID vs. non-COVID/LRTI**		
	**HR**	**95% CI**	**p** ** values**	**HR**	**95% CI**	**p** ** values**
COVID positive status	0.96	[0.79, 1.36]	0.131	2.09	[1.69, 2.76]	< 0.001
Age (years)	1.02	[0.99, 1.03]	0.063	1.04	[1.03, 1.05]	< 0.001
Male	1.07	[0.83, 1.38]	0.596	1.24	[1.52, 2.23]	< 0.001
Black vs. White	1.60	[0.99, 2.53]	0.051	1.19	[0.87, 1.63]	0.274
Other vs. White	1.25	[0.98, 2.51]	0.064	1.03	[0.76, 1.40]	0.859
Hispanic vs. non-Hispanic	0.82	[0.62, 1.09]	0.170	1.12	[0.96, 1.31]	0.165
HTN	1.79	[1.22, 2.61]	0.002	2.89	[1.85, 6.01]	< 0.001
Cardiovascular disease	1.71	[1.31, 2.23]	< 0.001	1.50	[1.01, 2.22]	0.020
Asthma	0.96	[0.71, 1.29]	0.777	1.06	[0.71, 1.59]	0.758
COPD	0.86	[0.62, 1.21]	0.402	0.90	[0.52, 1.59]	0.727
Obesity (or BMI > 30)	1.15	[0.89, 1.50]	0.288	0.84	[0.59, 1.19]	0.336

**Table 4:** Adjusted hazard ratios for newly diagnosed HTN and CKD among patients with T2D for (a) hospitalized COVID versus LRTI and (b) nonhospitalized COVID versus non-COVID/LRTI during 3-year follow-up in cohorts matched for age, sex, and ethnicity, adjusted for all comorbidities.

**HTN**	(A) Hosp COVID-19 vs. Hosp LRTI			(B) Non-hosp COVID vs. non-COVID/LRTI		
	AHR	95% CI	*p* values	AHR	95% CI	*p* values
COVID positive status	1.37	[0.76, 2.33]	0.398	1.74	[1.42, 2.19]	< 0.001

**CKD**	(A) Hosp COVID-19 vs. Hosp LRTI			(B) Non-hosp COVID vs. non-COVID/LRTI		
	HR	95% CI	*p* values	HR	95% CI	*p* values
COVID positive status	0.85	[0.63, 1.15]	0.187	2.02	[1.57, 2.60]	< 0.001

## Data Availability

The datasets used are available from the corresponding author upon reasonable request.

## Supporting Information

Supporting Information Additional supporting information can be found online in the Supporting Information section. Table S1. Type 2 diabetes OMOP concept ids. Table S2. LRTI and OMOP concept ids. Table S3. Demographics and pre-existing comorbidities at index date for matched (1:1) hospitalized COVID-19 versus hospitalized LRTI, and matched (1:1) nonhospitalized COVID-19 versus nonhospitalized LRTI. *p* value < 0.05 was considered significant between hospitalized COVID+ and LRTI patients and nonhospitalized COVID-19 patients and nonhospitalized COVID-19/LRTI negative patients. SD: standard deviation. Table S4. (A) Incidence and (B) HRs due COVID-19 status for developing new HTN and CKD for three different follow-up periods (2-6, 6-12, 12-36 months post index date). Table S5. Incidence due COVID-19 status for developing new HTN and CKD for three different time periods (2020–2021, 2021–2022, and 2022–2023).
