# Supplementary material for: Patients With Type 2 Diabetes Are at Greater Risk of Developing New Hypertension and Chronic Kidney Disease Following COVID-19
Source: J Diabetes Res. 2025 Jun 4;2025:8816198. doi: 10.1155/jdr/8816198 (PMC12158568; doi:10.1155/jdr/8816198)
Supplement: Supporting Information — Additional supporting information can be found online in the Supporting Information section. Table S1. Type 2 diabetes OMOP concept ids. Table S2. LRTI and OMOP concept ids. Table S3. Demographics and pre-existing comorbidities at index date for matched (1:1) hospitalized COVID-19 versus hospitalized LRTI, and matched (1:1) nonhospitalized COVID-19 versus nonhospitalized LRTI. p value < 0.05 was considered significant between hospitalized COVID+ and LRTI patients and nonhospitalized COVID-19 patients and nonhospitalized COVID-19/LRTI negative patients. SD: standard deviation. Table S4. (A) Incidence and (B) HRs due COVID-19 status for developing new HTN and CKD for three different follow-up periods (2-6, 6-12, 12-36 months post index date). Table S5. Incidence due COVID-19 status for developing new HTN and CKD for three different time periods (2020–2021, 2021–2022, and 2022–2023). [file 8816198.f1.docx]

**Supplemental Table 1.** Type 2 Diabetes OMOP concept ids

| 201530 | Hyperosmolar coma due to type 2 diabetes mellitus |
| --- | --- |
| 201826 | Type 2 diabetes mellitus |
| 376065 | Disorder of nervous system due to type 2 diabetes mellitus |
| 443729 | Peripheral circulatory disorder due to type 2 diabetes mellitus |
| 443731 | Renal disorder due to type 2 diabetes mellitus |
| 443733 | Disorder of eye due to type 2 diabetes mellitus |
| 4063043 | Pre-existing type 2 diabetes mellitus |
| 4099651 | Type 2 diabetes mellitus with ulcer |
| 4140466 | Lumbosacral radiculoplexus neuropathy due to type 2 diabetes mellitus |
| 4193704 | Type 2 diabetes mellitus without complication |
| 4196141 | Arthropathy due to type 2 diabetes mellitus |
| 4221495 | Cataract due to diabetes mellitus type 2 |
| 4222415 | Mononeuropathy due to type 2 diabetes mellitus |
| 4222876 | Gangrene due to type 2 diabetes mellitus |
| 4228443 | Ketoacidotic coma due to type 2 diabetes mellitus |
| 36714116 | Hypoglycemic coma due to type 2 diabetes mellitus |
| 37016349 | Hyperglycemia due to type 2 diabetes mellitus |
| 37016768 | Autonomic neuropathy due to type 2 diabetes mellitus |
| 37017432 | Polyneuropathy due to type 2 diabetes mellitus |
| 43530656 | Nonproliferative retinopathy due to type 2 diabetes mellitus |
| 43530685 | Proliferative retinopathy due to type 2 diabetes mellitus |
| 43530690 | Foot ulcer due to type 2 diabetes mellitus |
| 43531010 | Pre-existing type 2 diabetes mellitus in pregnancy |
| 43531563 | Neuropathic arthropathy due to type 2 diabetes mellitus |
| 43531578 | Chronic kidney disease due to type 2 diabetes mellitus |
| 43531616 | Dermopathy due to type 2 diabetes mellitus |
| 45757363 | Hypoglycemia due to type 2 diabetes mellitus |
| 45757435 | Mild nonproliferative retinopathy due to type 2 diabetes mellitus |
| 45770830 | Macular edema and retinopathy due to type 2 diabetes mellitus |
| 45770881 | Moderate nonproliferative retinopathy due to type 2 diabetes mellitus |
| 45773064 | Traction detachment of retina due to type 2 diabetes mellitus |

**Supplemental Table 2.** LRTI and OMOP concept ids

| 8689 | Influenza due to unidentified influenza virus with other respiratory manifestations |
| --- | --- |
| 920135 | Acute bronchiolitis due to human metapneumovirus |
| 35207931 | Influenza due to other identified influenza virus with other respiratory manifestations |
| 35207933 | Respiratory syncytial virus pneumonia |
| 35207934 | Parainfluenza virus pneumonia |
| 35207935 | Human metapneumovirus pneumonia |
| 35207937 | Viral pneumonia, unspecified |
| 35207938 | Pneumonia due to Streptococcus pneumoniae |
| 35207939 | Pneumonia due to Hemophilus influenzae |
| 35207940 | Pneumonia due to Klebsiella pneumoniae |
| 35207941 | Pneumonia due to Pseudomonas |
| 35207942 | Pneumonia due to streptococcus, group B |
| 35207943 | Pneumonia due to other streptococci |
| 35207944 | Pneumonia due to Escherichia coli |
| 35207945 | Pneumonia due to other Gram-negative bacteria |
| 35207947 | Pneumonia due to other specified bacteria |
| 35207948 | Unspecified bacterial pneumonia |
| 35207949 | Chlamydial pneumonia |
| 35207950 | Pneumonia due to other specified infectious organisms |
| 35207951 | Pneumonia in diseases classified elsewhere |
| 35207952 | Bronchopneumonia, unspecified organism |
| 35207953 | Lobar pneumonia, unspecified organism |
| 35207956 | Pneumonia, unspecified organism |
| 35207957 | Acute bronchitis due to Mycoplasma pneumoniae |
| 35207962 | Acute bronchitis due to respiratory syncytial virus |
| 35207963 | Acute bronchitis due to rhinovirus |
| 35207965 | Acute bronchitis due to other specified organisms |
| 35207966 | Acute bronchitis, unspecified |
| 35207967 | Acute bronchiolitis due to respiratory syncytial virus |
| 35207968 | Acute bronchiolitis due to other specified organisms |
| 35207969 | Acute bronchiolitis, unspecified |
| 45533545 | Pneumonia due to Methicillin susceptible Staphylococcus aureus |
| 45557620 | Pneumonia due to Methicillin resistant Staphylococcus aureus |
| 45581841 | Influenza due to identified novel influenza A virus with other manifestations |
| 45586661 | Influenza due to unidentified influenza virus with unspecified type of pneumonia |
| 45596276 | Influenza due to identified novel influenza A virus with pneumonia |

**Supplemental Table 3.** Demographics and pre-existing comorbidities at index date for matched (1:1) hospitalized COVID-19 versus hospitalized LRTI, and matched (1:1) non-hospitalized COVID-19 versus non-hospitalized LRTI. p value <0.05 was considered significant between hospitalized COVID+ and LRTI patients and non-hospitalized COVID-19 patients and non-hospitalized COVID-19/LRTI negative patients. SD: Standard Deviation.

|  | Hosp COVID-19  N = 1180 | Hosp LRTI  N = 1180 | p value | Non-hosp COVID-19  N = 4962 | Non-Hosp COVID-19/LRTI negative  N = 4962 | p value |
| --- | --- | --- | --- | --- | --- | --- |
| Demographics |  |  |  |  |  |  |
| Age, mean (±SD) | 69.39 (13.66) | 69.39 (13.66) | 0.999 | 60.95 (14.43) | 60.96 (14.42) | 0.997 |
| Male | 542 (45.93%) | 544 (46.10%) | 0.967 | 1890 (38.09%) | 1889 (38.07%) | 0.999 |
| Hispanic | 567 (48.05%) | 566 (47.97%) | 0.999 | 2197 (44.28%) | 2198 (44.30%) | 0.999 |
| White | 134 (11.36%) | 120 (10.17%) | 0.388 | 513 (10.34%) | 598 (12.05%) | 0.007 |
| Black | 398 (33.73%) | 442 (37.46%) | 0.065 | 1718 (34.62%) | 1290 (26.00%) | <0.001 |
| Other | 648 (54.92%) | 618 (52.37%) | 0.231 | 2731 (55.04%) | 3074 (61.95%) | <0.001 |
| Pre-existing Comorbidities N (%) | |  |  |  |  |  |
| Hypertension | 1075 (91.10%) | 1087 (92.12%) | 0.414 | 4052 (81.66%) | 3297 (66.44%) | <0.001 |
| CKD | 615 (52.12%) | 646 (54.75%) | 0.216 | 1423 (28.68%) | 591 (11.91%) | <0.001 |
| Cardiovascular Disease | 498 (42.20%) | 565 (47.88%) | 0.006 | 1064 (21.44%) | 333 (6.71%) | <0.001 |
| Asthma | 345 (29.24%) | 393 (33.31%) | 0.037 | 1447 (29.16%) | 617 (12.43%) | <0.001 |
| COPD | 275 (23.31%) | 395 (33.47%) | <0.001 | 590 (11.89%) | 185 (3.73%) | <0.001 |
| Obesity, BMI>30 | 537 (45.51%) | 513 (43.47%) | 0.341 | 2287 (46.09%) | 1079 (21.75%) | <0.001 |
| 1-yr pre-index HbA1c, mean(SD) | 7.59 (2.87)  n = 613 | 7.11 (1.55)  n = 518 | <0.001 | 7.40 (1.83)  n = 3833 | 7.83 (2.02)  n = 3530 | <0.001 |

**Supplemental Table 4. (A)** Incidence and **(B)** HRs due COVID-19 status for developing new HTN and CKD for three different follow-up periods (2-6, 6-12, 12-36 months post index date)

| (A) |  | Hosp COVID-19 vs Hosp LRTI | | | Non-hosp COVID vs. non-hosp controls | | |
| --- | --- | --- | --- | --- | --- | --- | --- |
|  |  | COVID | LRTI | p | COVID | controls | p |
| New HTN | 2-6 months | 44/524 (8.40%) | 8/87 (9.20%) | 0.96 | 29/745 = 3.89% | 125/3833 = 3.75% | 0.445 |
|  | 6-12 months | 47/455 (10.33%) | 3/59 (5.08%) | 0.28 | 40/688 = 5.81% | 140/4047 = 3.46% | 0.004 |
|  | 12-36 months | 52/407 (12.78%) | 4/43 (9.30%) | 0.67 | 52/534 = 9.74% | 277/5092 = 5.44% | <0.001 |
| New CKD | 2-6 months | 59/2289 (2.58%) | 16/467 (3.43%) | 0.38 | 42/3088 = 1.36% | 57/9801 = 0.58% | <0.001 |
|  | 6-12 months | 58/1987 (2.92%) | 15/352 (3.69%) | 0.24 | 49/2686 = 1.82% | 63/10224 = 0.62% | <0.001 |
|  | 12-36 months | 123/1830 (6.72%) | 19/276 (6.88%) | 1.00 | 56/2031 = 2.76% | 188/12880 = 1.46% | <0.001 |

| (B) |  | Hosp COVID-19 vs Hosp LRTI | | | Non-hosp COVID vs. non-hosp controls | | |
| --- | --- | --- | --- | --- | --- | --- | --- |
|  |  | HR | 95% CI | p | HR | 95% CI | p-values |
| New HTN | 2-6 months | 0.87 | [0.37, 2.06] | 0.75 | 1.73 | [1.05, 2.86] | 0.032 |
|  | 6-12 months | 1.54 | [0.44, 5.44] | 0.49 | 2.07 | [1.29, 3.30] | 0.002 |
|  | 12-36 months | 1.66 | [0.53, 5.15] | 0.37 | 2.46 | [1.67, 3.64] | <0.001 |
| New CKD | 2-6 months | 1.03 | [0.89, 1.76] | 0.07 | 1.45 | [0.67, 2.75] | 0.384 |
|  | 6-12 months | 0.79 | [0.44, 0.99] | 0.13 | 1.87 | [1.23, 3.30] | <0.001 |
|  | 12-36 months | 0.69 | [0.39, 1.22] | 0.20 | 1.84 | [1.31, 2.26] | <0.001 |

|  |  | Hosp COVID-19 | Hosp LRTI | p | Non-hosp COVID | non-hosp controls | p-values |
| --- | --- | --- | --- | --- | --- | --- | --- |
| New HTN | 2020-2021 | 70/420 (16.67%) | 1/18 (5.56%) | 0.35 | 42/138 (30.43%) | 1395/8033 (17.37%) | <0.001 |
|  | 2021-2022 | 86/814 (10.57%) | 9/98 (9.18%) | 0.80 | 75/425 (17.65%) | 1523/13000 (11.72%) | <0.001 |
|  | 2022-2023 | 82/1157 (7.09%) | 9/166 (5.42%) | 0.53 | 123/1267 (9.71%) | 1059/14448 (7.33%) | 0.002 |
| New  CKD | 2020-2021 | 134/1096 (12.23%) | 13/104 (12.50%) | 0.94 | 48/466 (10.30%) | 1359/24310 (5.59%) | <0.001 |
|  | 2021-2022 | 188/2010 (9.35%) | 32/312 (10.26%) | 0.69 | 97/1304 (7.44%) | 1375/33527 (4.10%) | <0.001 |
|  | 2022-2023 | 200/2835 (7.05%) | 51/522 (9.77%) | 0.04 | 159/3319 (4.79%) | 995/33911 (2.93%) | <0.001 |

**Supplemental Table 5.** Incidence due COVID-19 status for developing new HTN and CKD for three different time periods (2020-2021, 2021-2022, 2022-2023)
